# New perspectives on *corpora amylacea* in the human brain

**DOI:** 10.1038/srep41807

**Published:** 2017-02-03

**Authors:** Elisabet Augé, Itsaso Cabezón, Carme Pelegrí, Jordi Vilaplana

**Affiliations:** 1 Secció de Fisiologia, Departament de Bioquímica i Fisiologia, Facultat de Farmàcia i Ciències de l’Alimentació, Universitat de Barcelona, Av. Joan XXIII 27-31, 08028 Barcelona, Spain; 2 Institut de Neurociències, Universitat de Barcelona, Barcelona, Spain; 3 CIBERNED Centros de Biomedicina en Red de Enfermedades Neurodegenerativas, Spain

## Abstract

*Corpora amylacea* are structures of unknown origin and function that appear with age in human brains and are profuse in selected brain areas in several neurodegenerative conditions. They are constituted of glucose polymers and may contain waste elements derived from different cell types. As we previously found on particular polyglucosan bodies in mouse brain, we report here that *corpora amylacea* present some neo-epitopes that can be recognized by natural antibodies, a certain kind of antibodies that are involved in tissue homeostasis. We hypothesize that *corpora amylacea*, and probably some other polyglucosan bodies, are waste containers in which deleterious or residual products are isolated to be later eliminated through the action of the innate immune system. In any case, the presence of neo-epitopes on these structures and the existence of natural antibodies directed against them could become a new focal point for the study of both age-related and degenerative brain processes.

*Corpora amylacea* (CA) are glycoproteinaceous structures that were first reported in the brains of elderly patients by J.E. Purkinje at a scientific conference held in Prague in 1837. Although extensively discussed around the beginning of the nineteenth century, they were not considered to be of any pathological significance for many years. It was not until recent decades, with the development of new techniques, that interest in their nature and their relation with certain diseases awakened^1^. CA usually increase in number with age in normal human brains, but they are also profuse in selected areas of the brain in several neurodegenerative conditions, including Alzheimer’s, Parkinson’s, Huntington’s and Pick’s diseases, multiple and hippocampal sclerosis, and in patients with temporal lobe epilepsy and focal cortical dysplasia^2^,^3^. While essentially constituted of glucose polymers, an extraordinary number of components mainly derived from neurons, oligodendrocytes and astrocytes have been proposed to accompany them^1^,^2^. However, the presence of several of these components remains controversial and some results seem to be inconsistent. It is in part due to the uncertainty regarding their composition that the origin and role of CA still remain unclear; and CA are probably the cerebral structures that have been considered in the most different ways over the years. CA have been considered as: merely post-mortem artifacts^4^; the result of a defect in glycogen metabolism^5^; protein precipitates of lymphatic or hematogenous origin^4^; accumulations of breakdown products from neurons and oligodendroglial cells^6^; aggregated remnants of degenerated neuronal cells^7^; conglomerations of interacting proteins from degenerating neurons and extravasated blood elements released after the breakdown of the blood–brain barrier^8^; structures formed from degenerating astrocytes^9^; and recently, as pathological structures related to fungal infections^10^. The presence of waste elements is a recurrent feature, and CA may be involved in the trapping and sequestration of potentially hazardous products^1^, or they may act as a system that cleans the central nervous system (CNS)^11^. In any case, such diverse interpretations have not allowed for a comprehensive overview of CA.

CA are positive to periodic acid-Schiff (PAS) staining due to their high polysaccharide content. They have been associated with some pathological polyglucosan bodies (PGBs) that appear with age in mouse brain and are frequently referred to as “PAS granules” because they are also stained in the PAS reaction^12^. Studies we recently performed on mouse PAS granules determined that these structures result from a degenerative process affecting astrocytic processes and their surrounding neuropil^13^. We found that during their formation, some epitopes emerge and these epitopes should be considered as neo-epitopes because they are not present in healthy structures^14^. We also observed that these neo-epitopes are recognized by natural IgM antibodies which, as they are natural, are present in the blood plasma of mice from birth and without prior contact with external antigens^15^. These results indicate that the organism permanently has antibodies prepared to react against the neo-epitopes that arise in PAS granules. We also found that the IgM antibodies that recognize these neo-epitopes are also present in the plasma of other mammal species^15^, which is in accordance with the fact that natural antibodies have been fixed by natural selection during evolution and are therefore interspecific. Meanwhile, we also observed that these natural IgM antibodies are present as contaminants in a high percentage (around 70%) of commercial antibodies originated from mouse, rabbit, goat or rat, and obtained from ascites or serum, being monoclonal or polyclonal, and even supplied as purified^14^,^15^. Since these contaminant IgMs are recognized by the majority of anti-IgG antibodies used as secondary antibodies in immunohistochemical studies, these IgMs are the cause of numerous cases of false positive immunostaining of PAS granules, and they therefore account for some inconsistencies in some of the theories concerning PAS granules^12^.

Taking all the above into account and based on certain similarities between CA in human brains and PAS granules in mouse brains, we hypothesized that CA in human brains would also contain neo-epitopes and that human plasma, as well as plasma from other mammal species, would contain natural IgMs directed against them. If this hypothesis is confirmed, a new perspective on and interpretation of CA will be opened up. Moreover, we also hypothesized that the presence of contaminant IgMs in commercial antibodies causes much false positive immunostaining of CA and hence, several conclusions drawn from some of the studies published to date are unfounded, making it necessary to revise the current view of these structures.

## Results

### CA contain neo-epitopes that can be recognized by plasma IgMs

**Figure 1:**
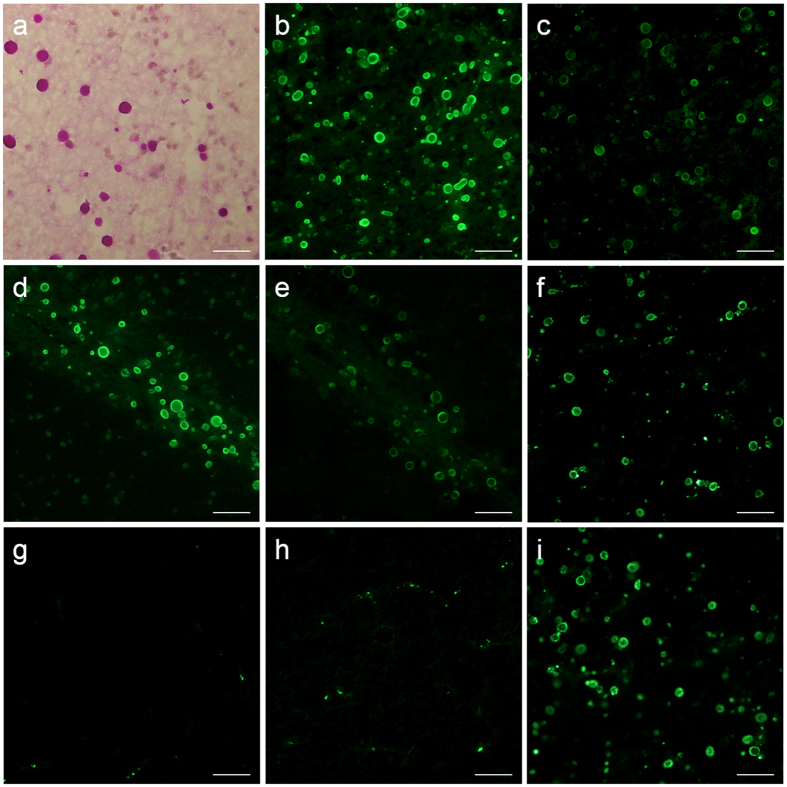
CA contain neo-epitopes that can be recognized by plasma IgMs. A representative image of PAS staining illustrating the presence of CA in a hippocampal brain section from an AD donor is shown in (**a**). Representative images from brain sections from an AD donor (**b** and **c**) and a control donor (**d** and **e**) immunostained with two different human blood plasma in the first incubation and anti-human IgM (μ chain specific) conjugated to a fluorochrome in the second incubation (**b** and **d** correspond to one plasma donor, and **c** and **e** to the other donor). As can be observed, CA were positively stained in all cases. If commercial purified human IgM antibodies were used in the first incubation, CA were also stained (**f**). Negative staining was observed when BB (**g**) and the Fc fragment of human IgM (**h**) were used in the first incubation, instead of human IgMs. Previous incubation with an Fc receptor blocker did not block the positive staining obtained when commercial purified human IgM was used in the first incubation (**i**). The last four representative images (**f–i**) were obtained from the same AD donor. Scale bar: 50 μm.

The presence of CA in brain hippocampal slices from Alzheimer’s disease (AD) and control donors was confirmed by carrying out standard PAS staining (Fig. 1a). Thereafter, we tested whether human plasma contains plasma IgMs directed against some components of the CA in these brains. We used brain slices from 3 AD and 3 control donors. From each brain donor, 8 brain slices were immunostained respectively with human blood plasma from 8 different donors in the first incubation and an anti-human IgM (μ chain specific) antibody conjugated to a fluorochrome in the second incubation. In all cases the CA become stained (Fig. 1b–e). Positive staining of CA was also observed when using commercial purified human IgM immunoglobulins in the first incubation (Fig. 1f). The absence of staining when using blocking buffer (BB) in the first incubation indicated that the IgMs are necessary for such staining (Fig. 1g). The presence of a receptor for the Fc region of the IgM in CA was ruled out on the basis of the absence of staining when using just the Fc fragment of the human IgM in the first incubation (Fig. 1h). Moreover, this was reinforced as previous incubation with a universal Fc receptor blocker did not block the staining with purified human IgM immunoglobulins (Fig. 1i). All these results suggest the presence of some neo-epitopes on CA that can be recognized by some human plasmatic IgMs.

### The IgMs that recognize the neo-epitopes contained on CA are natural IgMs

**Figure 2:**
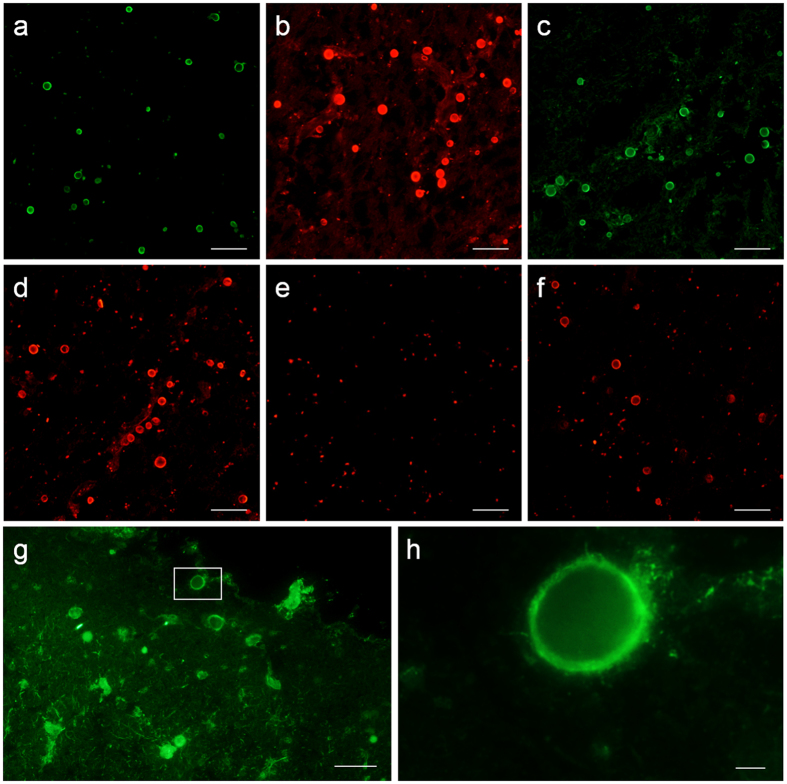
The IgMs that recognize CA neo-epitopes are natural antibodies. Representative images from an AD donor showing the positive staining of CA when using goat (**a**), rat (**b**), rabbit (**c**) and mouse (**d**) sera in the first incubation and an appropriate fluorochrome-labeled anti-IgM antibody in the second. Negative staining was obtained when BB was used in the first incubation instead of the different mammal sera and a representative image obtained when an anti-mouse IgM was used in the second incubation is shown (**e**). A representative image from the same AD donor showing the positive staining of CA with sera from SOPF mice is shown (**f**). Purified IgMs from umbilical cord sera positively stained the CA of an AD patient and a representative image is shown in (**g**). (**h**) Inset in (**g**). Scale bar (**a–g**) 50 μm; scale bar (**h**) 5 μm.

As natural IgMs are usually interspecific because their repertoire and reactivity pattern were determined by evolution, we checked for the existence of IgMs directed against the neo-epitopes of CA in sera from different mammal species. We used brain slices from 4 different AD brain donors. For each donor, 4 different brain sections were immunostained using sera from goat, rat, rabbit and mouse in the first incubation and the appropriate fluorochrome-labeled anti-IgM antibodies in the second. In all cases, CA become stained (Fig. 2a–d). Negative controls performed with BB in the first incubation and the different fluorochrome-labeled anti-IgM antibodies in the second did not stain CA (Fig. 2e), indicating that IgMs are essential for such staining. Therefore, the presence of IgMs directed against the neo-epitopes of CA in all the mammal species tested suggests that these IgMs are natural antibodies.

Meanwhile, taking into account that natural antibodies are present from birth, without any need for previous contact with external antigens, we also immunostained different brain sections from the 4 AD brain donors with sera obtained from mice maintained from birth in specific opportunistic pathogen free (SOPF) conditions. In all cases, CA became stained, indicating that also in SOPF conditions mice can contain IgMs that recognize some CA components (Fig. 2f), and reinforcing that these IgMs are in fact natural antibodies.

We also tested whether the IgMs directed against the neo-epitopes of CA were also contained in sera obtained from human umbilical cords. It must be pointed that, unlike IgGs, IgMs cannot cross the placenta and those contained in serum from the umbilical cord are produced by the fetus and are considered natural antibodies^16^. We immunostained different brain slices from an AD donor with purified IgMs obtained from 3 different umbilical cord sera in the first incubation and human anti-IgM antibodies in the second; and CA become stained in all cases (Fig. 2g-h). Moreover, as indicated before, the staining was not present when using BB in the first incubation and human anti-IgM antibodies in the second. These results strongly support the finding that the IgMs that recognize some components of CA are natural antibodies.

### The IgMs that recognize the neo-epitopes contained in CA do not reach these neo-epitopes *in vivo*

As indicated earlier, when using BB in the first incubation and fluorochrome-labeled anti-human IgM specific antibody in the second, there was no positive staining of CA in any of the slices tested from the different brain donors (Fig. 1g). As natural antibodies are permanently present in the plasma of organisms, and consequently it can be assumed that they were also present in the plasma of the brain donors, we can deduce that in neither AD or control patients had the plasma IgMs reached the CA. This observation is equivalent to that made when studying the PAS granules of mice, in which we were able to demonstrate that all mice with granules in their hippocampus contained IgMs directed against the neo-epitopes in their plasma; but that *in vivo*, and due to the blood–brain barrier, these IgMs do not reach the PAS granules in any of these animals^15^.

### The anti-neo-epitopes IgMs can cause false positive staining of CA

**Figure 3:**
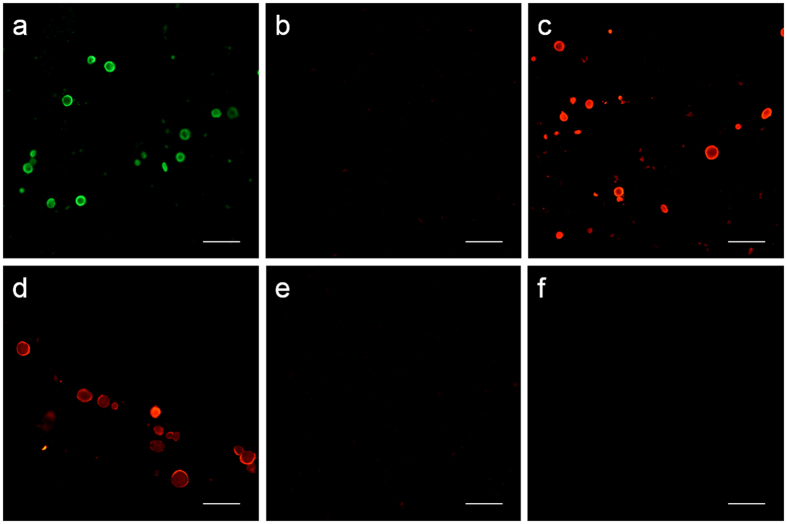
IgMs can produce false positive staining of CA. Representative images of brain sections from an AD patient stained with JJ319 mouse IgG_1_ primary antibody and: a secondary fluorochrome-labeled antibody against heavy and light chains of mouse IgG (**a**) a secondary fluorochrome-labeled antibody against the γ_1_ chain of mouse IgG (**b**) and a secondary fluorochrome-labeled antibody against the μ chain of mouse IgM (**c**). As can be observed, CA are not stained when the secondary antibody against the γ_1_ chain of mouse IgG is used. When purified IgMs or purified IgGs from JJ319 antibody were used in the first incubation (**d** and **e** respectively) and a fluorochrome-labeled antibody against the μ chain was used in both cases in the second incubation, positive staining of CA was obtained when using purified IgMs while it was negative when using purified IgGs. Negative staining of CA was also obtained when purified IgGs were incubated followed by a secondary antibody against heavy and light chains of mouse IgG (**f**). It can therefore be concluded that only IgM bind to the CA and that the staining when using a secondary antibody against heavy and light chains of mouse IgG is just a cross-reaction with IgMs. Scale bar: 50 μm.

In our previous studies on PAS granules in mice, we observed that a high number of commercial antibodies contain contaminant natural IgMs that produce false immunostaining of them^14^. We therefore checked whether contaminant IgMs also produce false positive immunostaining of CA, and the results obtained confirmed that they do. One example of such false positive staining can be observed when using the JJ319 mouse IgG_1_ primary antibody directed against rat CD28 surface antigen of lymphocytes, with no reactivity in brain, but containing contaminant IgMs. The immunostaining of CA contained in hippocampal sections from 4 different AD donors with the JJ319 primary antibody and a secondary fluorochrome-labeled antibody against heavy and light chains of mouse IgG appeared to be positive in all cases (Fig. 3a). This positive staining, however, is just a misinterpretation. CA were not stained when using the specific anti-γ-chain of mouse IgG_1_ as the secondary antibody, indicating the absence of IgG_1_ bound to the CA (Fig. 3b). The staining was positive when using a secondary antibody directed against the μ-chain of mouse IgM, indicating the presence of contaminant IgMs bound to the CA (Fig. 3c). The staining with secondary antibodies directed against the μ-chain of mouse IgM remained when using the IgM fraction of the JJ319 in the first incubation (Fig. 3d), whereas it disappeared when using its IgG fraction in the first incubation (Fig. 3e). Finally, the staining of CA with the IgG fraction in the first incubation also failed when using the secondary fluorochrome-labeled antibody against heavy and light chains of mouse IgG (Fig. 3f). We can therefore conclude that the initial staining observed when using the unpurified JJ319 antibody in the first incubation and the secondary antibody directed against heavy and light chains of mouse IgG was simply due to the cross-reaction of this secondary antibody with the contaminant IgMs bound to the CA. As the secondary antibodies used in immunohistochemical studies are usually directed against heavy and light chains, misinterpretation due to the presence of contaminant natural IgMs could be frequent and could explain some of the contradictory results regarding the composition of CA.

### CA do not contain tau protein or amyloid–β peptides

Some contradictory results concerning the composition of CA are related to their content of tau protein or the presence of amyloid-β protein precursor (AβPP) or their derived amyloid-β (Aβ) peptides^3^,^17^,^18^,^19^,^20^,^21^,^22^. Therefore, we revised the immunostaining of CA using different commercial antibodies directed against these components. Taking into account the possible presence of contaminant IgMs, we ruled out the use of secondary antibodies directed against the heavy and light chains of IgGs, because they can cross-react with the IgMs. Instead, we used secondary antibodies directed against the γ_1_-chain specific for IgG_1_ and secondary antibodies directed against the μ-chain specific for IgMs.

**Figure 4:**
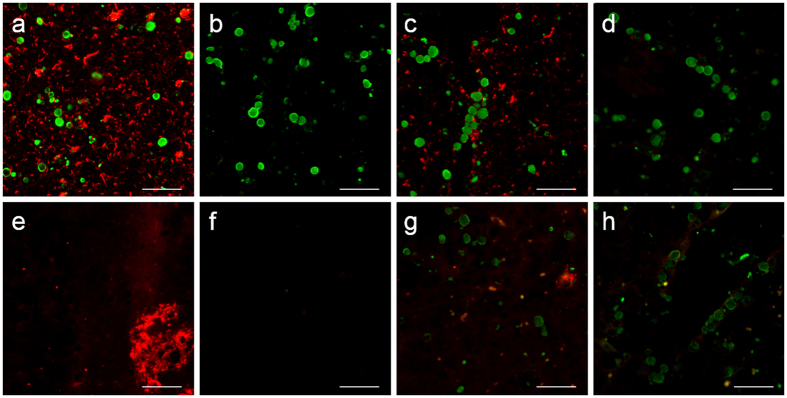
CA do not contain tau protein or amyloid–β peptides. Representative images obtained from brain samples from an AD donor stained with: anti-tau clone tau 5 (**a**) pre-adsorbed anti-tau clone tau 5 (**b**) anti-tau clone tau 5E2 (**c**) and pre-adsorbed anti-tau clone tau 5E2 (**d**), followed by both fluorochrome-labeled secondary antibodies directed against the μ chain of mouse IgM (green) and against the γ_1_ chain of mouse IgG (red). When using non-pre-adsorbed antibodies, CA were positively stained with the contaminant IgMs contained in the anti-tau antibodies (green) and neurofibrillary tangles and neurons were stained with the anti-tau IgG_1_ (red) (**a** and **c**). When the antibodies were pre-adsorbed with tau protein, the staining of the neuro fibrillary tangles and neurons disappeared while that of CA by contaminant IgMs remained (**b** and **d**). Representative images obtained from brain samples from an AD donor stained with: the 6E10 antibody (**e**) pre-adsorbed 6E10 (**f**) the 12F4 antibody (**g**) and pre-adsorbed 12F4 (**h**), followed by both fluorochrome-labeled secondary antibodies directed against the μ chain of mouse IgM (green) and against the γ_1_ chain of mouse IgG (red). When using non-pre-adsorbed anti-Aβ IgG_1_ antibodies, amyloid plaques appeared positively stained with IgGs (red) (**e** and **g**). CA did not stain after 6E10 staining because this antibody did not contain contaminant IgM (**e**) while they appeared stained with 12F4 (green), denoting contamination of IgMs in this antibody (**g**). When the antibodies were pre-adsorbed with Aβ fragments, the staining of the amyloid plaques disappeared while that of CA by contaminant IgMs remained (**h**). Scale bar: 50 μm.

In order to check the presence of tau, we used an anti-tau protein IgG_1_ antibody (clone Tau5) and brain slices from 4 AD donors. When the second incubation contained both the antibody against the μ-chain (green fluorescence) and the antibody directed against the γ_1_-chain (red fluorescence), we observed positive staining of CA corresponding to the μ-chain but not to the γ_1_-chain, indicating that the vial contained contaminant IgMs that bind to the CA, but that anti-tau IgG_1_ does not bind to them. We also observed that neurofibrillary tangles were positively stained with the IgGs but not with the IgMs, denoting the presence of tau protein and the absence of neo-epitopes recognized by IgMs in these pathological structures (Fig. 4a). Consistent with this, when the anti-tau antibody was pre-adsorbed with tau protein, the staining of the neurofibrillary tangles with IgG disappeared; but that of the IgM on CA did not (Fig. 4b). The same procedure was repeated with another primary antibody directed against the tau protein (clone 5E2), and the results were reproduced exactly (Fig. 4cand d). It can therefore be concluded that CA do not contain tau protein, at least not at levels that are detected by our immunostaining procedures.

The presence of AβPP or some of their derived Aβ peptides on CA was also checked with two different primary antibodies in brain slices from 4 different AD donors. We used one primary antibody directed against the 1–16 region of the Aβ_42_ (clone 6E10), which also recognizes the AβPP, and another primary antibody directed against Aβ_42_ (clone 12F4) which only recognizes the Aβ_42_ peptide. In all cases, the secondary incubations contained both the anti-γ_1_-chain (red fluorescence) and the anti-μ-chain (green fluorescence) antibodies. The 6E10 antibody, which did not contain IgM contamination, indicated the presence of Aβ in amyloid plaques but not in CA (Fig. 4e). Moreover, the positive staining of amyloid plaques disappeared when the primary antibody was pre-adsorbed with Aβ protein (Fig. 4f). The antibody 12F4 corroborated the presence of Aβ on amyloid plaques and its absence in CA. In this case, however, the vial contained contaminant IgMs that bound to CA but not to amyloid plaques (Fig. 4g). Now, when pre-adsorbing the primary antibody with Aβ protein, the positive staining of the amyloid plaques disappeared; while the staining of CA with IgM was maintained (Fig. 4f). All together, these results indicate that CA do not contain AβPP or Aβ peptides, at least not at levels that are detected by our immunostaining procedures.

## Discussion

The most important outcome of the present work is the presence of natural IgM antibodies in human plasma that are capable of recognizing some neo-epitopes on human brain CA. This conclusion is derived from the different sequential findings. In the first place, the fact that human plasma from different donors are capable of immunostaining CA when using fluorochrome-labeled anti-IgMs as the secondary antibodies suggests that there are IgMs in human plasma that recognize some of their components. Secondly, as plasma contains more than IgMs, we subsequently checked for the staining with purified IgMs to rule out some possible staining artifacts, and positive staining was also observed. Moreover, we observed that the Fc fragments of the IgMs are insufficient to stain the CA and we verified that a universal Fc receptor blocker does not block the staining with IgMs. Thus, the staining with the IgMs seems to be related to the variable regions of the IgMs, which are those that contain the antigen-binding sites. In order to determine if the IgMs are natural antibodies, we used different approaches. The most noteworthy was the use of purified IgMs obtained from sera of human umbilical cords. As IgMs do not cross the placenta, these IgMs are generated by the fetus before exposure to external antigens, and are part of the repertoire of natural antibodies. We observed that IgMs from umbilical cords can stain CA. Moreover, IgMs that recognize some CA components are also found in sera from mammals other than human, which is consistent with the fact that natural antibodies are often interspecific. We also observed that they are present in sera from mice maintained from birth in SOPF conditions, without any contact with opportunistic pathogens. Altogether, these sequential findings reveal a connection between the natural immune system and CA, and seem to indicate some useful aspects concerning CA.

Natural antibodies, which can act as a first line of defense against external elements, also play an important role in the maintenance of tissue homeostasis. They act, among others, on neo-epitopes that appear in situations of cellular stress and tissue damage; but also even during conventional tissue cell turnover^23^,^24^,^25^. Taking into account that CA may be involved in the trapping and sequestering of potentially hazardous products^1^, the presence of neo-epitopes in CA could just be a consequence of the presence of some altered components in them, such as oxidized lipids like phosphorylcholine or the oxidation-associated determinant malondialdehyde, which can become an adduct on proteins^23^,^26^. However, not only are CA a random accumulation of several components, but they are also concretions or compact structures with a high glucidic content and often with a periventricular or perivascular location. This could be related to their extrusion from brain tissue. CA seem to be elements in an organized process and in fact, they have been considered as a part of a physiological cleaning system of the CNS^11^. The existence of natural antibodies directed against the neo-epitopes located on CA seems to be congruent with their possible pre-programed elimination. The clearance of dying cells is one of the most essential roles of the natural immune system and, in mouse, natural IgM antibodies that recognize apoptotic cells have been shown to enhance the phagocytic clearance of these cells without triggering harmful inflammatory responses in the tissue^23^,^27^. The enhancement of phagocytosis processes mediated by natural IgMs and the presence of neo-epitopes on CA suggest that these structures may be phagocyted, which is in agreement with the potential cleaning mechanism attributed to CA. Reinforcing this possibility, it should be pointed out that the presence of CA in the brain does not correlate with local inflammatory processes and moreover, in *neuromyelitis optica*, CA located in the injured regions are collected and engulfed by infiltrating macrophages^9^.

As we have shown in this study, these IgMs directed against the neo-epitopes of CA are widely present in human plasma and in serum from umbilical cord, as well as in the serum of different species of mammals, including animals kept in SOPF conditions. Together, this indicates that these antibodies are directed against ubiquitous antigens. By using antigen microarray informatics, it has been determined that IgM natural antibodies are directed to a specific and limited set of self-molecules that are highly constant between newborns^16^,^28^. However, the number of molecules studied was limited, and it is still unknown in which specific structures these interactions occur *in vivo* or what the exact function of this self-reactivity against each one of these molecules would be. To the best of our knowledge, little is known about natural antibodies directed against neo-epitopes located on CNS structures. One study reported two human monoclonal natural antibodies targeting myelin and cell-surface antigens on oligodendrocytes; those antibodies seemed to promote the remyelination of neuronal fibers^29^,^30^. Other studies proposed the presence of natural antibodies targeting Aβ peptides in the serum and cerebrospinal fluid of healthy individuals^31^,^32^. However, these last findings were not confirmed by our present studies, in which we did not observe natural antibodies against amyloid plaques in samples from AD donors. Based on the components assumed to be present in CA, we can conjecture as to the possible nature of the neo-epitopes contained in them. However, given the false positive staining that the contaminant IgMs present in commercial antibodies has produced in immunohistochemical studies of CA, there is a need to revise their composition. As we point out in this work, it will be necessary to revise the papers that consider the presence of tau and Aβ in CA; and there is also a need to clarify the presence of other doubtful components, such as nestin^33^, alpha-synuclein^20^,^21^,^33^, GFAP^3^,^21^,^33^,^34^ or some Heat-shock proteins like HSP70^33^,^35^,^36^,^37^. There is, nonetheless, general consensus as to the accumulation of waste products in CA^1^,^2^,^3^ and the presence of ubiquitin in these structures^3^,^19^,^20^,^35^,^38^. It should be pointed out that ubiquitin is associated with mechanisms for the cleaning of waste products, which reinforces the idea that CA are organized structures that can eliminate waste products. Consistently with this, PAS granules from mice, which have a high glucidic content and seem to contain waste products from different cellular origins, also contain ubiquitin^39^,^40^.

In the CNS, the presence of the blood–brain barrier generally restricts the access of plasma IgMs to the brain parenchyma and, as we describe herein, in AD and control donors, the IgMs do not have access to the neo-epitopes present in CA. However, as indicated above, in the case of *neuromyelitis optica*, in which the blood–brain barrier is altered in the injured regions, the CA are collected and engulfed by infiltrating macrophages^9^. Meanwhile, some studies indicate that CA can be extruded from the marginal glia at the vestibular root entry, and it has been proposed that they could be a component of the glio-pial system of clearance that can remove different molecules from the CNS^11^. Taken as a whole, the binding between IgMs and CA could take place inside the CNS when the blood–brain barrier is altered or outside the CNS after CA extrusion.

**Figure 5:**
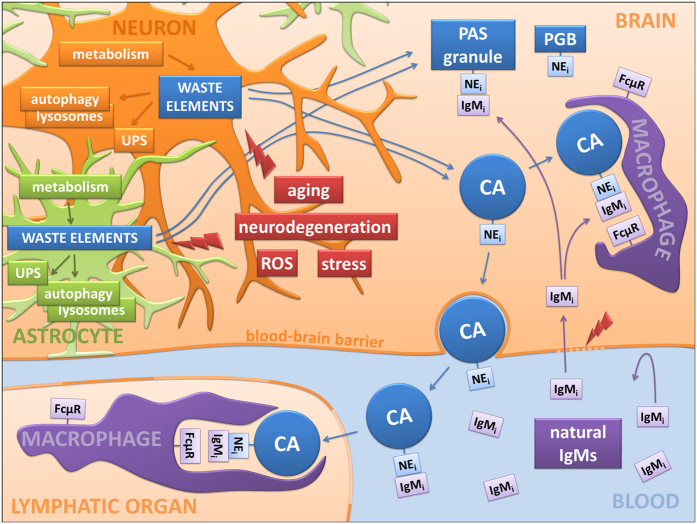
Schematic integrative proposal. Waste elements generated from neurons, astrocytes, oligodendrocytes and other brain cells are removed intracellularly via the ubiquitin proteasome system (UPS) or via autophagic processes linked to lysosomal digestion. However, some of these waste elements can be found in polyglucosan bodies (PGBs) like CA in human brains or PAS granules in mouse brains, mainly when the production of waste elements is strengthened by factors such as aging, neurodegenerative disorders, reactive oxygen species or cellular stress. These PGBs contain some neo-epitopes (NEi) that are targeted by natural antibodies of the IgM type (IgMi). Since in physiological conditions the blood –brain barrier prevents access of IgMs to the brain parenchyma, the linkage between IgMs and CA is produced outside the brain after CA extrusion or inside the brain if the barrier is disrupted, thus allowing the passage of the IgMs. Macrophages, which contain specific receptors for the activated IgMs (FcμR), can subsequently phagocytize them. The new relationship shown here between PGBs and the natural immune system suggests that these PGBs could be waste containers ready to be eliminated by the action of this predetermined immune system.

Taking all the above into account, the picture obtained supports the idea that CA are waste containers involved in protective or cleaning mechanisms, in which potentially deleterious or residual cellular products arising through different processes and in different cell types are sequestered to be later eliminated via phagocytic processes or other mechanisms in which the innate immune system plays an important role (Fig. 5).

In the present work we highlight a new link between CA and the innate immune system, and this link will certainly help to ascertain the nature and functions of CA. Moreover, as the neo-epitopes that are target of natural IgMs have also been observed in PAS granules in mice^12^,^15^, these properties could perhaps be expanded to other PGBs, making it necessary to study the presence of neo-epitopes in other related brain degenerative bodies such as Lafora bodies from Lafora disease or Bielschowsky bodies from Bielschowsky body disease. In the same way in which the presence of neo-epitopes in certain malignant tumor cells and the presence of IgMs directed against these neo-epitopes is a recent field of study in the therapy and diagnosis of cancerous processes^25^,^41^,^42^, so the existence of neo-epitopes on CA and other degenerative brain structures and the presence of natural IgM antibodies directed against them could become a new focus of the study of age-related or pathological degenerative brain processes. It may open up new lines of research into PGBs and their physiopathological roles.

## Methods

### Human brain samples and blood

Post-mortem brain samples were purchased from the Banc de Teixits Neurològics (Biobanc-Hospital Clínic-IDIBAPS, Barcelona). Brain samples were taken from seven cases of neuropathologically confirmed Alzheimer’s disease patients (A3B3C3 stage, 73–92 years old) and three non-AD patients (79–95 years old, referred as control donors). On arrival at the laboratory, frozen hippocampal sections (6 μm) were stored at −80 °C. The number of brain samples used in each staining is indicated in the result section. For the most part, a minimum of three AD and three control brain donors were used for each specific staining.

Human blood samples (n = 8) collected with K3-EDTA were obtained from healthy donors from the Banc de Sang i Teixits de Barcelona. The samples were centrifuged at 12,000 rpm for 5 min in a Biofuge Pico centrifuge (Heraeus, Madrid, Spain) at 4 °C and plasma was collected and stored at −20 °C until use.

Foetal blood samples (n = 3) were obtained from the Maternal/Fetal Medicine Biobanc, I + D Fetal Medicine Research Center (IDIBAPS).

Rabbit and goat sera were obtained from Jackson ImmunoResearch Laboratories (Newmarket, UK) and rat, mouse and mouse SOPF sera were obtained from our laboratory as described previously^15^.

All methods involving animals and human samples were performed in accordance with appropriate guidelines and regulations. All experiments involving human tissue were approved by the Bioethical Committee of the University of Barcelona and all the animal experimental procedures were reviewed and approved by the University of Barcelona Animal Experimentation Ethics Committee (DAAM 7504).

### Periodic acid-Schiff staining

Brain sections were stained with PAS according to the standard procedure. Briefly, sections were fixed for 10 min in Carnoy’s solution (60% ethanol, 30% chloroform and 10% glacial acetic acid). Then the slides were pretreated for 10 minutes with 0.25% periodic acid (19324–50, Electron Microscopy Sciences) in distilled water followed by a washing step for 3 min. Then the slides were immersed in Schiff’s reagent (26052–06, Electron Microscopy Sciences) for 10 min. After the Schiff’s reagent, the slides were washed for 5 min in distilled water. Nuclei were counterstained for 6 min with a hematoxylin solution according to Mayer (3870, J.T. Baker, Center Valley, USA). Then the slides were washed, dehydrated to xylene and coverslipped with quick-mounting medium (Eukitt, Fluka Analytical, Germany).

### Antibodies and reagents

The following antibodies were used as primary antibodies: human IgM purified immunoglobulins (1:2; OBT1524; AbD Serotec, Kidlington, UK), mouse monoclonal IgG_1_ against Aβ peptide 1–42 (12F4; 1:100; SIG-39142; Covance, Princeton, USA), mouse monoclonal IgG_1_ anti-Aβ 1–16 (6E10; 1:50; SIG-39320; Covance), mouse monoclonal IgG_1_ anti-tau, a.a.210–241 (clone Tau-5; 1:100; MAB361; Merck Millipore, Darmstadt, Germany), mouse monoclonal IgG_1_ anti-tau (clone 5E2; 1:50; 05–348; Merck Millipore) and mouse polyclonal IgG_1_ anti-rat CD28 (JJ319; 1:50; kindly provided by Prof. T. Hünig). The IgM fraction of JJ319 was obtained from JJ319 using an MBP agarose column (PierceTM IgM Purification Kit; Thermo Scientific, Rockford, IL, USA), and the IgG fraction of JJ319 was obtained using a protein A column. Human adult sera were used as the primary antibodies at 1:100 dilution. Rabbit, rat and goat sera were used at 1:50 dilution, while mouse and SOPF mouse sera were pooled (n = 3, respectively, ICR-CD1, 3 months) and used at 1:100 dilution. The IgM fractions from human umbilical cord sera were purified from umbilical cord sera also with MBP agarose column, and were concentrated using the Pierce Protein Concentrators PES, 30 K MWCO (Thermo Scientific).

The following antibodies were used as secondary antibodies: AF488 goat anti-human IgM heavy chain (1:200; A-21215; Life Technologies, Eugene, OR, USA), AF594 goat anti-rat IgM heavy chain (1:250; A-21213; Life Technologies), rabbit anti-goat IgM conjugated to fluorescein isothiocyanate (FITC) (1:250; ab112861; Abcam, Cambridge, UK), goat anti-rabbit IgM μ chain-FITC (1:250; ab98458; Abcam), goat anti-mouse IgM μ chain specific conjugated to tetramethyl rhodamine-isothiocyanate (TRITC) (1:250; 115–025–020; Jackson ImmunoResearch Laboratories), AF488 goat anti-mouse IgM μ chain specific (1:250; 115–545–075; Jackson ImmunoResearch Laboratories), AF594 goat anti-mouse IgG_1_ (1:250; A-21125; Life Technologies) and AF488 donkey anti-mouse IgG (H + L) (1:250; A-21202; Life Technologies).

### Immunohistochemistry

For the immunohistochemistry, human brain sections were air dried for 10 min and then fixed with acetone for 10 min at 4 °C. After 2 h of drying, sections were rehydrated with phosphate-buffered saline (PBS) and then blocked and permeabilized with 1% bovine serum albumin in PBS (Sigma-Aldrich) (Blocking buffer, BB) with 0.1% Triton X-100 (Sigma-Aldrich) for 20 min. They were washed with PBS and the primary incubation was overnight at 4 °C. Afterwards, the slides were washed and incubated for 1 h at room temperature with the secondary antibody. Nuclear staining was performed with Hoechst (2 μg/mL, H-33258, Fluka, Madrid, Spain) and the slides were washed and coverslipped with Fluoromount (Electron Microscopy Sciences, Hatfield, PA, USA). Negative controls of staining were performed using BB in the first incubation instead of the primary antibodies. Pre-adsorption controls for tau and Aβ immunostaining were performed by incubating the antibodies overnight at 4 °C with mild agitation with a 10–20 molar-fold concentration of tau protein (AG952; Merck Millipore), Aβ protein fragment 1–42 (A9810; Sigma, Saint Louis, Missouri) or Aβ 1–40 (AS-24235; Anaspec, Fremont, CA). To rule out the presence of Fc receptors in CA, a universal Fc receptor blocker (NB309–15; Innovex Bioscience, Richmond, CA) was incubated before the incubation of the human IgMs, and the staining with Natural Human IgM Fc fragment protein (1:2; ab90287; Abcam) was also tested in the first incubation instead of complete IgMs.

### Image acquisition

Images were captured with a fluorescence laser and optical microscope (BX41, Olympus, Germany) and stored in tiff format. All the images were acquired using the same microscope, laser and software settings. The time of exposition was adapted to each staining, but the respective control images were acquired with the same time of exposition. Image analysis and treatment were performed using the ImageJ program (National Institute of Health, USA). Images that were modified for contrast and brightness to enhance their visualization were processed in the same way as the images corresponding to their respective controls.

## Additional Information

**How to cite this article**: Augé, E. *et al*. New perspectives on *corpora amylacea* in the human brain. *Sci. Rep.* **7**, 41807; doi: 10.1038/srep41807 (2017).

**Publisher's note:** Springer Nature remains neutral with regard to jurisdictional claims in published maps and institutional affiliations.
